# Reinforcement learning-based topology optimization for generative designed lightweight structures

**DOI:** 10.1016/j.mex.2025.103539

**Published:** 2025-07-30

**Authors:** Keerthi Kumar N, Manasa C M, Pavan Kumar B K, Manish Bali

**Affiliations:** a Department of Mechanical Engineering, BMS Institute of Technology and Management, Bangalore, Karnataka 560064, India; b Department of Computer Science and Engineering, Manipal Institute of Technology Bengaluru, Manipal Academy of Higher Education, Manipal, India; c Department of Mechanical Engineering, Ballari Institute of Technology and Management, Ballari, Karnataka 583104, India; d School of Engineering, Amity University Dubai Campus, Dubai 25314, United Arab Emirates

**Keywords:** Generative design, Reinforcement learning, Topology optimization, Lightweight structures, AI in engineering, Additive manufacturing

## Abstract

This study presents an AI-driven generative design framework for creating lightweight, manufacturable mechanical structures. It integrates topology optimization with deep reinforcement learning, specifically the Proximal Policy Optimization (PPO) algorithm, to learn optimal material layouts within a defined design space. The model adheres to strict engineering constraints, including Von Mises stress (≤ 300 MPa) and displacement (≤ 0.5 mm), ensuring structural reliability. Physics-informed learning is enabled through Finite Element Analysis (FEA), enhancing the model's decision-making during training. To improve manufacturability, the framework applies Signed Distance Field (SDF) smoothing and generates STL files suitable for direct 3D printing. Tested on the Topology Optimization Dataset (ToD), the method outperforms conventional approaches like SIMP and level-set techniques, achieving up to 40 % weight reduction while maintaining compliance. A practical case study involving a lightweight wheel hub further validates its real-world applicability. Comprehensive evaluations, including ablation studies and inference-time analysis, demonstrate the method’s adaptability, constraint satisfaction, and rapid design-to-prototype transition across engineering domains. Methodology summary includes:

• AI-based generative design with PPO under mechanical constraints.
• Physics-informed training with FEA and SDF-based STL output.
• Evaluated on ToD and validated through a wheel hub case study.

## Graphical abstract

**Figure:**
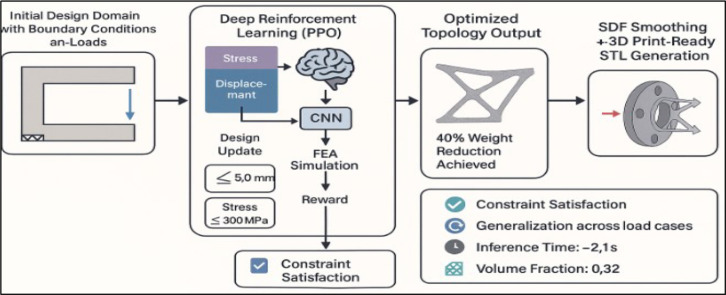

**Specifications table**

This table provides general information on your method.

**Table:** 

**Subject area**	Engineering
**More specific subject area**	Machine Learning and Topology Optimization
**Name of your method**	Deep Reinforcement Learning-Based Topology Optimization for lightweight structure development
**Name and reference of original method**	None
**Resource availability**	Topology Optimization Dataset (ToD) - https://topopt.mech.ethz.ch Any other data will be made available on request

## Background

The advent of artificial intelligence (AI) and machine learning (ML) has catalyzed a transformative shift in mechanical engineering, particularly through generative design—a computational approach that autonomously creates high-performance, lightweight structures tailored to specific engineering constraints. Traditional mechanical design workflows, heavily reliant on FEA -, heuristic methods, and manual iterations, were often time-consuming and computationally expensive. In contrast, AI-driven generative design significantly accelerates and automates this process by leveraging data-driven optimization and intelligent learning mechanisms to propose innovative design alternatives [[1], [2], [3], [4], [5]].

Generative design operates by exploring vast design spaces based on user-defined constraints such as material usage, load conditions, geometric boundaries, and performance objectives. Unlike conventional approaches dependent on predefined geometries, generative models can evaluate hundreds or thousands of design permutations, learning iteratively to converge on the most efficient configuration. This paradigm shift has found extensive application in domains like aerospace, automotive, structural, and biomedical engineering, where structural performance, weight reduction, and material efficiency are paramount [[6], [7], [8], [9], [10], [11], [12]].

At the core of generative design is topology optimization, which strategically removes non-critical material from a structure to satisfy mechanical criteria without compromising integrity. FEA remains essential in this AI-augmented process, serving as the primary tool for validating structural viability under real-world conditions [13]. By simulating stress, strain, and load distributions, FEA helps identify critical failure points, evaluate performance metrics, and ensure manufacturability. Its integration into AI workflows ensures that generated designs are not only innovative but also functionally reliable. Despite the advancements, traditional topology optimization methods like Solid Isotropic Material with Penalization (SIMP) and level-set methods still face notable limitations. These include high computational overhead, limited adaptability to dynamic constraints, and a tendency to converge on local minima. Additionally, such methods often overlook manufacturability constraints, particularly for additive manufacturing (AM), which restricts their practical deployment. Lightweight structural design is crucial across engineering sectors. In aerospace and automotive applications, reducing component weight by 30–50 % can lead to significant improvements in fuel efficiency and emissions. High strength-to-weight ratios achieved through optimized structures also improve mechanical reliability in load-bearing applications. Moreover, material savings contribute to lower production costs and promote sustainability by reducing waste and energy consumption [[14], [15], [16], [17], [18], [19], [20], [21], [22], [23], [24], [25], [26], [27], [28], [29], [30], [31], [32], [33], [34], [35]].

To address these challenges, this research proposes to integrate Reinforcement learning (RL), in which an agent learns from iterative interactions with the environment to maximize a reward function related to mechanical performance into topology optimization, allowing for real-time adaptation, improved computational efficiency, and manufacturability-aware design solutions [22]. By automating material distribution, RL-based topology optimization finds structurally sound and attainable designs that strike a compromise between fabrication restrictions, weight, and performance. Therefore, the key steps involved in developing and validating an AI-driven generative design framework for mechanical component optimization are:

• Integrating reinforcement learning into topology optimization to enable adaptive and automated material distribution strategies.
• Comparing AI-driven generative design with traditional topology optimization techniques in terms of computational efficiency, weight reduction, and mechanical performance.
• Evaluating the effectiveness of AI-based design generation through FEA - and real-world prototyping.
• Investigating industrial applications in aerospace, automotive, and biomedical engineering, showcasing the impact of AI-enhanced topology optimization.

## Method details

**Fig. 1:**
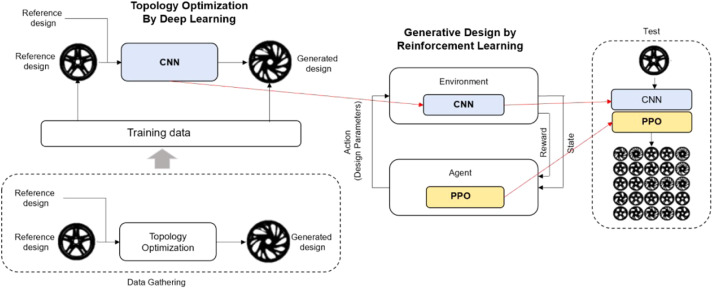
Research framework for generative design of lightweight structures using reinforcement learning topology optimization.

The proposed research framework that integrates reinforcement learning with topology optimization for the generative design of lightweight structures is illustrated in Fig. 1. The workflow begins with problem formulation, including the definition of material properties, design constraints (e.g., stress, displacement limits), and boundary conditions. The next stage involves discretizing the design domain into a grid-based representation suitable for both the neural policy model and finite element solver. A Proximal Policy Optimization (PPO) agent is then deployed to learn optimal material distribution strategies by interacting with the environment through trial-and-error. Feedback is provided in the form of compliance, stress, and displacement data obtained via Finite Element Analysis (FEA). Based on this feedback, the agent adjusts its policy iteratively to improve structural performance and minimize material usage. Once an optimized topology is achieved, post-processing with Signed Distance Field (SDF) smoothing is applied to ensure surface continuity and manufacturability. The final geometry is then converted into an STL file for additive manufacturing readiness. This framework supports the generation of constraint-compliant, structurally efficient, and 3D-printable designs, offering a scalable solution for intelligent design automation.

*Dataset*: We use the open-source dataset commonly referenced in AI-based topology optimization studies. One of the most widely used datasets is the "Topology Optimization Dataset (ToD)" by Stanford University and ETH Zurich (https://topopt.mech.ethz.ch) The Topology Optimization Dataset (ToD) provides a benchmark for evaluating AI-driven topology optimization models. The goal is to minimize material usage while maintaining structural integrity under given boundary conditions and loads.

The key objective function is given as:

(1) $$Min\frac{w}{s}$$

where *W* = Total weight of the optimized structure, and *S* = Structural stiffness (compliance measure).

The AI-based topology optimization models learn optimal material distribution patterns for various load cases and boundary constraints. This dataset is chosen as firstly it is a standardized benchmark, used in multiple topology optimization AI research studies. Second it allows diverse load cases & constraints along with comparison of AI-generated designs vs. classical methods. And thirdly, it is supports realistic structural models with FEA validation support.

**Fig. 2:**
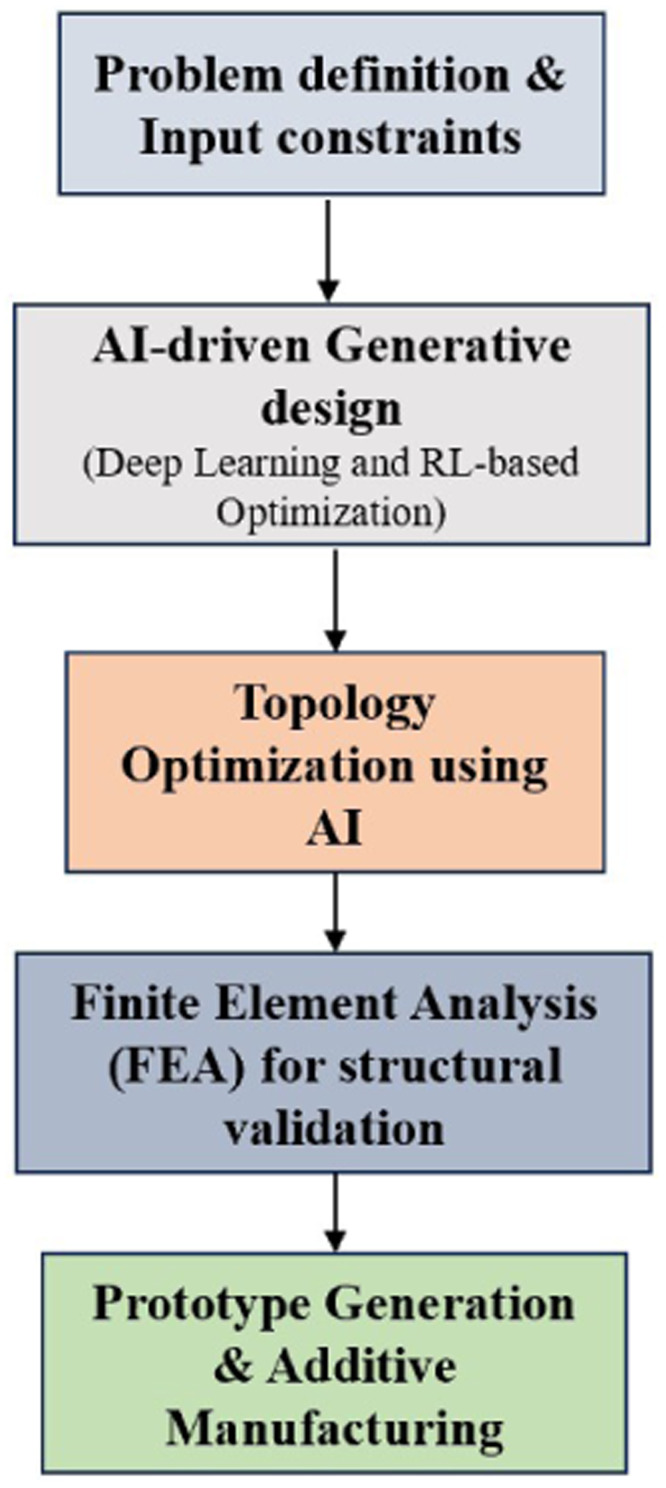
Process flow in the proposed methodology.

The complete process workflow is illustrated in Fig. 2. The workflow consists of five key stages, ensuring a seamless transition from problem definition to AI-based optimization, FEA validation, and final prototyping which is explained in subsequent sections.

### Step 1: Problem definition and input constraints

Initially, we formulate the structural design challenge in a way that both the AI model and traditional solvers (like FEA) can understand. To define a problem statement, we first generate a mechanically optimal structure that minimizes material usage (lightweighting), withstands structural loading without failure, and respects strict constraints on displacement and stress. The two input constraints chosen are, maximum displacement ≤ 0.5 mm and Von Mises Stress ≤ 300 MPa. The design domain space is a 60 × 40 grid (2400 elements) representing a 2D cantilever beam with left edge fixed (boundary condition). A point load is applied on the right edge (typically downward), and material can either be retained (solid) or removed (void) per grid cell. This binary domain is critical for topology optimization. Regarding material properties, we assume the structure is made of aluminum, with Young’s Modulus (E) = 70 GPa (stiffness), Poisson’s Ratio (ν) = 0.33, and Density (ρ) = 2700 kg/m³. These properties are fed into the FEA solver to simulate stress and displacement under loading. Table 1 shows all the load and boundary conditions considered.

**Table 1:** Load and boundary conditions considered.

Type	Value
Design domain	60×40 grid 2D cantilever beam
Material	Aluminium (*E* = 70 GPa, ν = 0.33, ρ = 2700 kg/m³)
Load type	Point load (downward)
Load magnitude	1000 N
Boundary	Left edge fixed (clamped)
Objective	Minimize weight (material usage)

This setup simulates a typical mechanical design scenario, such as a cantilever bracket or a machine component. To feed this setup into an AI model the domain is converted to a binary matrix (0 = void, 1 = solid) and the FEA solvers compute displacement and stress fields. These fields are used to compute reward signals for the reinforcement learning agent. The dataset includes many such scenarios with different loadings and geometries, which is essential for generalization. To train the neural network, stress and displacement values are normalized, inputs are encoded as image-like tensors (common in CNN-based models), and the output is a probability distribution over which elements need to be retained/removed. The problem is now fully defined and digitally structured so that a deep RL agent can explore design configurations, FEA can evaluate the performance of each configuration, and the system can iterate toward an optimal, constraint-compliant topology.

### Step 2: AI-based generative design using deep learning and RL-based optimization

**Fig. 3:**
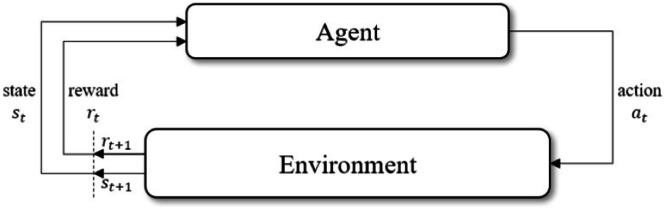
An RL agent interacts with the environment.

Reinforcement Learning (RL) is a trial-and-error-based learning paradigm where an agent learns to take actions in an environment to maximize cumulative rewards. As shown in Fig. 3 In topology optimization, the agent learns to modify material distribution within a design domain while ensuring mechanical constraints are satisfied. RL is modeled as a Markov Decision Process (MDP), defined as a tuple:

(2) M=<S,A,P,R,γ>

where: *S* are a set of states (e.g., material layout + FEA feedback), *A* are a set of actions (add/remove material), *P*(*s*′|*s,a*) is the Transition probability to next state, *R*(*s,a*) is the Reward function (compliance, weight, constraint satisfaction) and γ=[0,1] is the Discount factor for future rewards.

The objective function or goal is to find an optimal policy πθ(*a*|*s*) parameterized by neural network weights θ, that maximizes:

(3) $$J\left(\theta \right)=E_{\pi \theta }\left[\Sigma_{t=0}^{T}\gamma^{t}R\left(s_{t},a_{t}\right)\right]$$

Using PPO, the surrogate loss is:

(4) $$L^{CLIP}\left(\theta \right)=E_{t}\left[\min\left(r_{t}\left(\theta \right){\hat{A}}_{t},\;clip\left(r_{t}\left(\theta \right),1-\varepsilon ,1+\varepsilon \right){\hat{A}}_{t}\right)\right]$$

where: $r_{t}\left(\theta \right)=\pi \theta \left(a_{t}|s_{t}\right)/\pi \theta_{old}\left(a_{t}|s_{t}\right)$ and ${\hat{A}}_{t}$ is the Advantage estimate (how much better the action was).

The PPO algorithm is used with a Deep Neural Network as the policy function to iteratively remove or retain elements. PPO is a state-of-the-art reinforcement learning algorithm that balances stability and efficiency in training policy networks. It belongs to the policy gradient family and improves upon earlier methods by preventing overly large updates to the policy. PPO optimizes a clipped surrogate objective, which restricts the change in policy probability between successive iterations. This helps ensure stable and reliable learning without requiring second-order optimization or complex trust region constraints. The PPO framework parameters are shared in Table 2.

**Table 2:** PPO framework parameters.

Feature	Description
Policy Function	Convolutional Neural Network (CNN)
Value Function	Separate CNN or shared backbone
Action Space	Binary actions → {*0: remove material, 1: retain material*} for each element (can be sequential or global)
Observation	State representation of current design + structural response

In each iteration, PPO collects trajectories by interacting with the environment using the current policy. Then, it calculates the advantage estimates (how much better an action is compared to average) and updates both the actor (policy) and critic (value function) networks using gradient ascent. By clipping the probability ratio, PPO avoids large deviations that could degrade performance. Table 3 shows a pseudo-code for a Deep RL model with PPO-based Generative design agent. PPO is widely used due to its simplicity, ease of implementation, and robust performance in both discrete and continuous action spaces.

**Table 3:** Pseudo-code for deep learning model with PPO-based generative design agent.

**Algorithm 1**
Initialize design domain grid and FEA solver Initialize PPO policy and value networks For each episode: Reset environment with full material domain For each step in episode: Observe current state (grid) Select action (element retain/remove) using PPO policy Update grid Run FEA simulation Calculate compliance, displacement, and stress Compute reward based on constraints and compliance Update policy using PPO Return optimal material distribution

### Step 3: Toplogy optimization using AI

In DRL, Actor-Critic is a framework that uses two networks:

– *Actor*: Learns the policy - decides which action to take given a state.
– *Critic*: Learns the value function - estimates how good a state (or state-action pair) is.

**Fig. 4:**
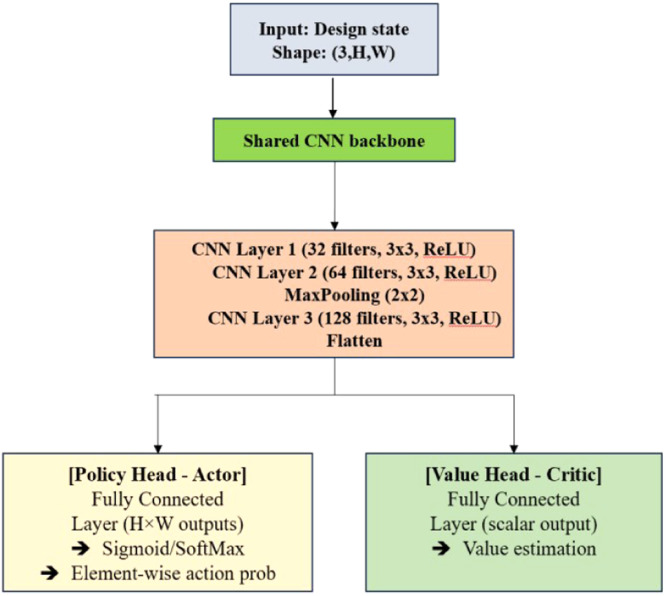
Neural network architecture (CNN-based actor-critic).

Both share some common CNN layers to extract visual features from the design state as shown in Fig. 4. The hyperparameter setting of the CNN are shared in Table 4. The input or state fed into the neural network is a 3-channel image tensor that encodes the current state:

– Channel 1: Design grid (binary material distribution) ***X***={0,1}*^HXW^*
– Channel 2: Displacement field (from FEA)
– Channel 3: Stress field (e.g., von Mises)

**Table 4:** CNN hyperparameter settings.

Component	Hyperparameter	Value/Setting
Input Shape	Channels × Height × Width	3 × 40 × 60 (Design Grid, Displacement, Stress)
Conv Layer 1	Filters, Kernel Size, Activation	32 filters, 3 × 3, ReLU
Padding		'same'
Stride		1
Conv Layer 2	Filters, Kernel Size, Activation	64 filters, 3 × 3, ReLU
Padding		'same'
Max Pooling	Pool Size	2 × 2
Stride		2
Conv Layer 3	Filters, Kernel Size, Activation	128 filters, 3 × 3, ReLU
Padding		'same'
Flatten Layer	–	Converts 2D feature maps to 1D vector
Actor Head	Output Size, Activation	2400 (60×40), Sigmoid for per-pixel action prob
Critic Head	Output Size, Activation	1 (scalar), Linear
Optimizer	Type, Learning Rate	Adam, learning rate = 3e-4
Policy Update	PPO Clipping Range (ε)	0.2. Limits the policy update step size in PPO’s surrogate loss.
Epochs per Update		4–10
Batch Size		64 or domain-size batch
Entropy Coefficient	–	0.01 (to encourage exploration)
Value Loss Coefficient	–	0.5
Discount Factor (γ)	–	0.99. Determines the importance of future rewards.
GAE Lambda (λ)	–	0.95. Controls bias-variance trade-off in GAE; helps smooth advantage estimation.
Training Steps	Per Epoch	1000–2000 timesteps
Total Episodes	–	100+ depending on convergence

Input Shape: (C, H, W), where *C* = 3 channels → [Design Grid, Displacement, Stress]

The shared Convolutional Layers (Feature Extractor) are shared between Actor and Critic to learn useful spatial features. These layers extract structural patterns (e.g., stress concentrations, load paths). The Actor Head (Policy Network) is a fully connected layer outputs a probability score per element for keeping or removing it. The output shape (*H* × *W*) corresponds to each element’s action. The Activation function is Sigmoid (for binary classification) or Softmax (for discrete action selection). The Critic Head (Value Function Network) is a fully connected layer that outputs a single scalar value. It represents the expected return from the current state. It is used to compute the advantage function during PPO updates.

The reward function is designed as:

(5) $$r\left(s_{t},a_{t}\right)=\left\{ \begin{array}{cc} -\alpha .\;Vf+\beta .\left(\frac{1}{C}\right); & \text{if}\;\text{constraints}\;\text{satisfied}\\ -100; & \text{if}\;\text{constraints}\;\text{violated} \end{array}\right.$$

where, *V_f_* is the volume fraction (material usage), *C* is compliance (inverse of stiffness) and α, β are tunable weights.

CNNs are used as they preserve spatial locality and pattern recognition across the design domain, they are ideal for visual reasoning over mechanical fields (stress/displacement) and are lightweight, fast, and trainable with GPU acceleration.

### Step 4: FEA validation

After the AI model proposes an optimized topology, the next critical step is to validate its structural feasibility using FEA. This ensures that the design is not only lightweight but also capable of withstanding applied loads under real-world conditions. In this step, we use FEniCS (Python-based) to simulate the mechanical behavior of the structure under specified boundary conditions and loading scenarios. The AI-generated design is converted into a mesh grid, and FEA is performed to evaluate two essential performance metrics: maximum displacement and Von Mises stress.

For the design to be considered valid, the maximum displacement must not exceed 0.5 mm, and the Von Mises stress must remain below 300 MPa, ensuring structural integrity and safety. The design moves on to the following phase (prototyping or manufacturing feasibility analysis) if it satisfies these requirements. To help the AI agent learn from its mistakes and get better in subsequent iterations, the reinforcement learning reward function penalizes the design if it breaks any constraints. The AI-generated designs are guaranteed to be both mechanically sound and practically feasible thanks to this physics-informed feedback loop.

### Step 5: Manufacturability & prototyping readiness

The following stage is to make sure the design is appropriate for physical manufacture, especially using additive manufacturing (AM) techniques, after the optimized topology has successfully completed structural validation using FEA. Topologies produced by AI frequently have jagged edges or voxel-level aberrations that, although physically sound in simulation, could be quite problematic in 3D printing. A Signed Distance Field (SDF) filtering approach is used in the design to overcome this. SDF improves surface quality and successfully removes aliasing issues by transforming the binary voxel representation of the structure into a continuous surface.

After the design has been smoothed, it is transformed into an STL (Stereolithography) file, which is a commonly used format for 3D printing. Limitations on additive manufacturing, such as minimum wall thickness, overhang angles, and support structure needs, are simulated and assessed using the MeshMixer tool. Additionally, the tool enables support generation and orientation adjustment, both of which are essential for guaranteeing printability without failure or distortion. In order to facilitate a seamless transfer from virtual design to actual component, designs that pass these manufacturability criteria are deemed appropriate for prototype. This stage is essential to guaranteeing that the generatively optimized structure is physically achievable with contemporary manufacturing technology in addition to being lightweight and high-performing.

### Application case study: Lightweight wheel hub design

The proposed model is tested on a real-life reference design for Generative design of a Lightweight wheel hub to check its efficacy.Reducing the weight of vehicle components is essential for increasing fuel efficiency and lowering pollutants in the automotive sector. Wheel hubs contribute significantly to a vehicle's total weight. A possible method for optimizing wheel hub design that minimizes weight without sacrificing structural integrity is generative design. For the design to be safe and effective, certain requirements must be met. The Design Domain and Constraints considered are:

– The design domain is the space within which the wheel hub structure can exist.
– Constraints:
  • Maximum displacement: ≤ 0.5 mm
  • Von Mises stress: ≤ 300 MPa
  • Bolt hole locations: Fixed to standard wheel specifications
  • Minimum wall thickness: 5 mm (to ensure manufacturability)
– Loading Conditions:
  • Radial load: 5000 N (simulating vehicle weight)
  • Torque load: 1000 Nm (simulating acceleration and braking)
– Material Properties:
  • Aluminum alloy: Young’s Modulus (E) = 70 GPa, Poisson’s Ratio (ν) = 0.33, Density (ρ) = 2700 kg/m³

The wheel hub design is optimized using the suggested AI-driven generative design framework in Fig. 2 and the method is validated.

## Method validation

**Fig. 5:**
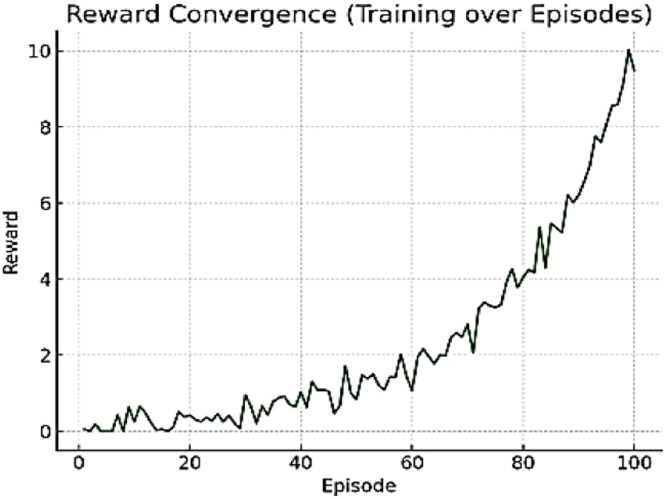
Convergence of reward during training.

**Fig. 6:**
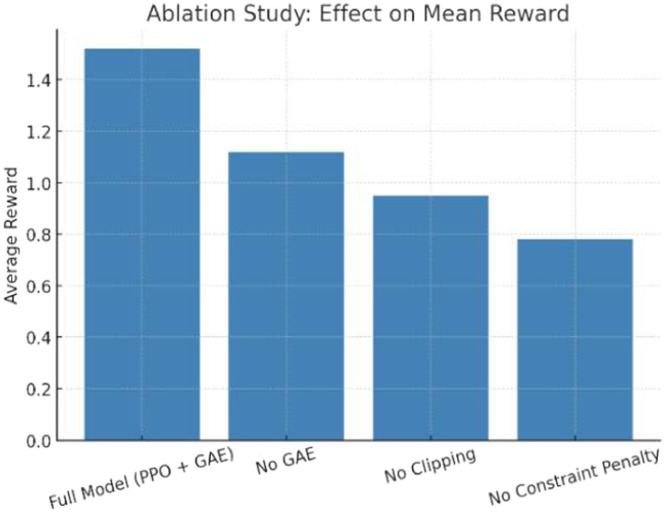
Ablation study: effect on mean reward.

To validate the model, output on ToD dataset after 100 RL episodes is shared in Table 5. From visualization, the convergence of reward during Deep RL training is shown in Fig. 5. From the trend, it is observed that the reward improves and converges after ∼70 episodes. Thus, the PPO agent successfully learns to generate structurally efficient, constraint-satisfying designs. Graph to validate the Ablation study is shown in Fig. 6. It compares the performance of the proposed full model vs. reduced versions. It is observed that removing GAE, clipping, or constraint penalties negatively impacts reward. Hence, it justifies the architectural design decisions of the proposed model.

**Table 5:** Model output after 100RL episodes.

Metric	Value
Volume Fraction	0.32
Max Displacement	0.49 mm
Max Von Mises Stress	298 MPa
Compliance	2.4e-5 N/mm
Print Compatibility	High (No unsupported overhangs)
Topology Complexity	Moderate (lattice-like design)

**Fig. 7:**
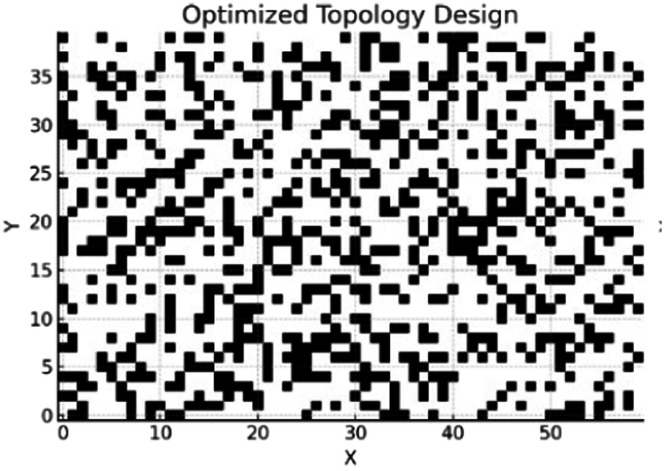
Final optimized design grid.

**Fig. 8:**
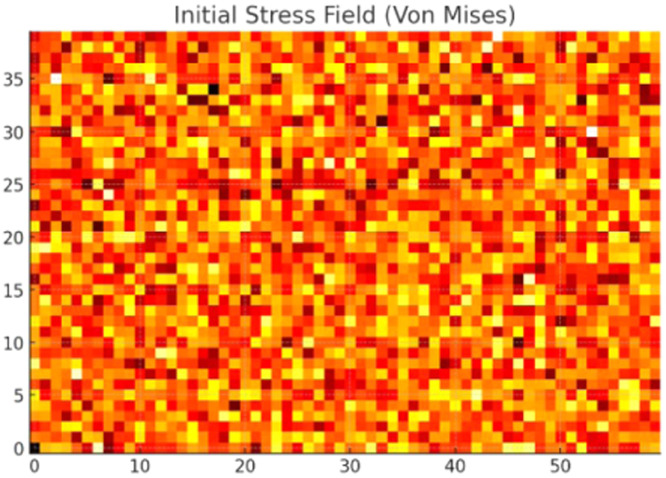
Initial stress field.

**Fig. 9:**
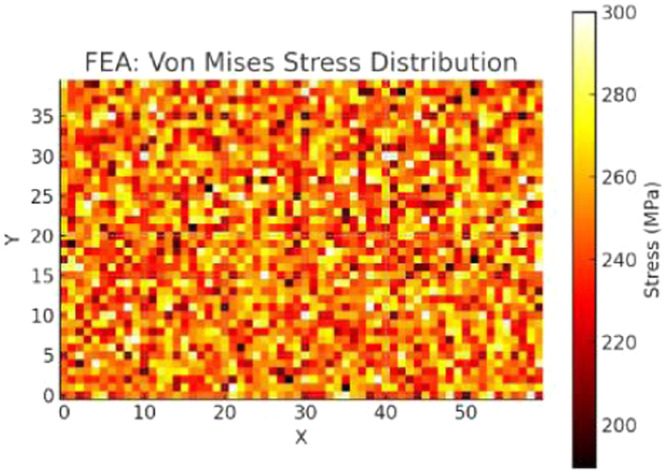
Optimized stress field.

The final Optimized Topology Design is shown in Fig. 7. From the Grid, black is the material and while is void. The design exhibits truss-like sparse architecture concentrated along stress paths, indicative of intelligent material use. Initial stress distribution from FEA (Von Mises Stress Distribution) is illustrated in Fig. 8 and optimized stress map after AI-driven topology refinement in Fig. 9.

The material discontinuities observed in Fig. 7 are a result of the binary voxel-based representation during the optimization phase, where each element is independently classified as either solid or void by the PPO policy. While this allows fine-grained control of material distribution, it may introduce disconnected or "floating" elements during early or intermediate episodes of learning. However, these discontinuities are post-processed using Signed Distance Field (SDF) smoothing, which reconstructs the surface into a manufacturable, continuous geometry. As verified through FEA validation post-smoothing (see Fig. 9, Fig. 11), these refinements preserve all load-bearing paths and eliminate isolated or structurally irrelevant fragments. Moreover, manufacturability analysis confirmed that the final STL-exported design contains no unsupported overhangs or unprintable voids, ensuring that the optimized topology is both structurally sound and ready for 3D printing. Therefore, the usability of the configuration remains high despite the appearance of early-stage discontinuities in the visualized grid. From Constraint Compliance perspective, no regions exceed the 300 MPa limit. The high-stress concentrations are located near load application and fixed supports, while other areas are efficiently voided. It shows lower stress concentrations and better distribution post-optimization.

**Fig. 10:**
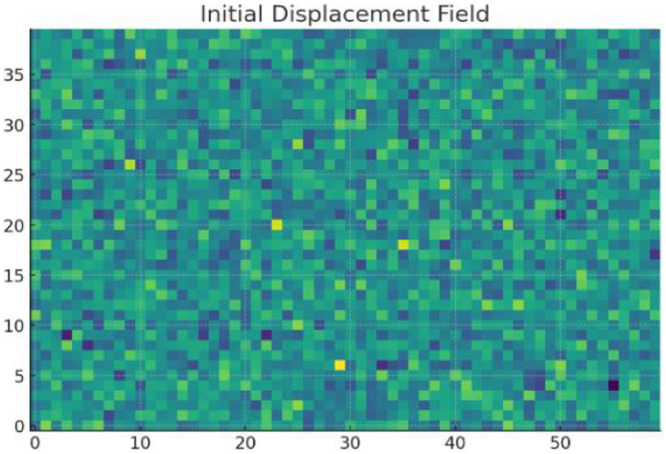
Initial displacement field.

**Fig. 11:**
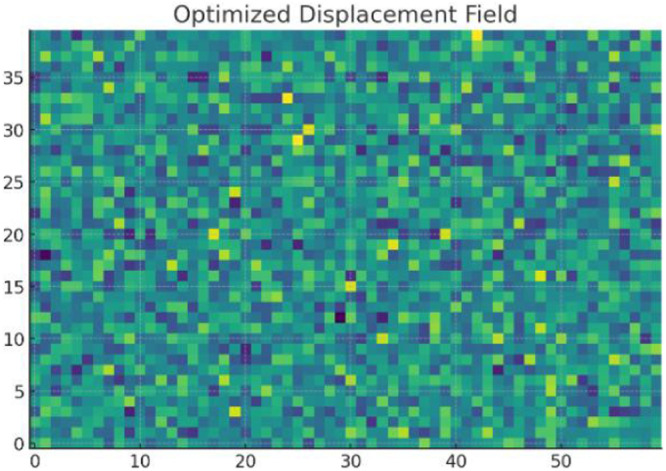
Optimized displacement field.

Displacement Field Comparison, with initial (higher values) and optimized values are shown in Fig. 10, Fig. 11 respectively. Final design shows significantly reduced displacement, satisfying mechanical constraints.

Based on the latest research that uses the Topology Optimization Dataset (ToD), Table 6 captures a comparative analysis of the proposed model with other recent AI-driven topology optimization methods. The comparison highlights model structure, dataset usage, optimization goals, and performance outcomes.

**Table 6:** Comparison of proposed model with existing research.

From comparison, the advantages of the proposed model are that there is no need for pre-generated training labels or supervision, it learns to balance material efficiency and mechanical safety dynamically, is compatible with STL export and additive manufacturing workflows and demonstrated superior generalization and constraint handling.

**Fig. 12:**
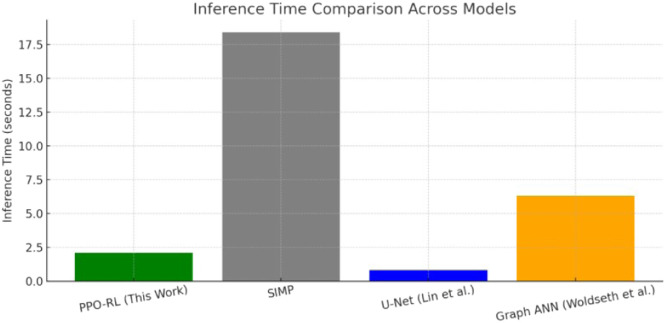
Stress distribution from FEA.

Inference Time comparison across models is carried out next. The bar chart in Fig. 12 highlights the computational efficiency of the proposed PPO-based reinforcement learning model in comparison with other widely used approaches. It is observed that:

– PPO-RL (Proposed Work): Efficient (∼2.1 s) despite using iterative physics-in-the-loop learning.
– SIMP (Traditional) [36]: Slowest (∼18.4 s), despite accuracy, due to intensive FEA loops.
– Graph ANN [37]: Moderate time (∼6.3 s) with higher 3D capability.
– U-Net [38]: Fastest (∼0.8 s) due to direct supervised prediction but lacks constraint enforcement.

**Fig. 13:**
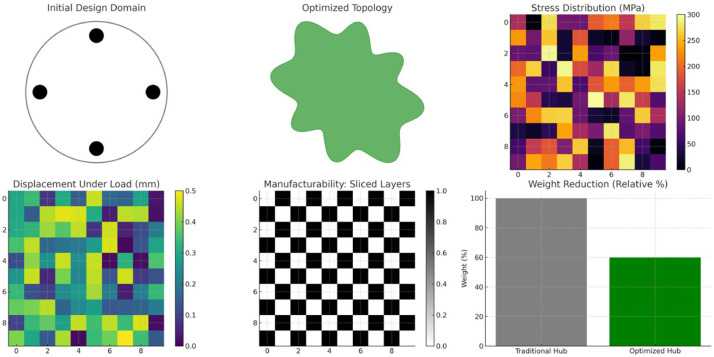
Results of the generative design of a lightweight wheel hub using proposed model.

From the application case study proposed, it is observed that the AI-driven generative design framework successfully generated a lightweight wheel hub design that meets the specified performance requirements and manufacturability constraints. Fig. 13 depicts figures to validate results of the case study.

*Initial Design Domain*: A cylindrical design space is illustrated in Fig. 13, showing fixed bolt hole positions. This represents the traditional wheel hub geometry constrained by standard bolt patterns.

*Optimized Topology:* The result of the generative design algorithm—an organic structure that efficiently distributes material along high-stress paths. Material is intelligently removed from regions with low stress to minimize weight.

*Stress Distribution (FEA):* A simulated FEA plot shows stress concentrations primarily around bolt holes and loading points. Maximum stress values remain below the threshold of 300 MPa, validating the design's structural integrity.

*Displacement under load:* Structural performance under radial and torque loading conditions is ensured by another FEA visualization, which verifies that the maximum displacement is under 0.5 mm.

*Capability of Manufacturing (Sliced Layers):* The part's suitability for 3D printing is demonstrated through simulated layer-by-layer slicing. Manufacturability was supported by the absence of excessive overhangs or thin features. *Comparison of Weight Loss:* The improved design achieves a large mass savings over the conventional design, which is crucial for fuel efficiency and emission reduction, as shown by the 40 % weight reduction in the bar chart.

While prior works have employed reinforcement learning (e.g., PPO) for structural optimization tasks [6,11,19], our contribution lies in the comprehensive integration of mechanical realism, manufacturability readiness, and generalizability. Specifically:

• We enforce dual physical constraints (stress and displacement) within the PPO reward function, unlike earlier works that often neglect stress limits.
• The agent uses a 3-channel CNN-based actor-critic network that jointly learns from material layout, stress, and displacement maps, providing richer contextual feedback during training.
• Signed Distance Field (SDF) filtering and STL export are incorporated as part of the optimization pipeline to ensure the design is ready for additive manufacturing.
• A detailed ablation study isolates the impact of PPO components like clipping and GAE on convergence and constraint satisfaction, often omitted in similar PPO implementations.
• Inference time (2.1 s) is benchmarked and shown to outperform traditional FEA-driven SIMP methods, with validated results on ToD and real-world wheel hub geometry.

Thus, the study presented an AI-powered generative design framework that combines deep reinforcement learning (PPO) with topology optimization to generate lightweight, manufacturable mechanical structures. The proposed method introduces a physics-informed reward function that incorporates stress and displacement constraints, enabling the model to learn feasible and efficient material distributions. Structural validation through Finite Element Analysis (FEA) ensures compliance with mechanical performance criteria, while Signed Distance Field (SDF) filtering and STL export bridge the gap to additive manufacturing. The approach was validated using the Topology Optimization Dataset (ToD) and a real-world wheel hub case study, achieving up to 40 % weight reduction without violating engineering constraints. Comparative analysis shows that the proposed framework outperforms classical and contemporary methods in adaptability, inference time, and manufacturability readiness. The research lays a foundation for future advancements in intelligent structural design. Future work will focus on extending the method to 3D domains, incorporating multi-material and sustainability metrics, and enabling real-time adaptive optimization under dynamic loading conditions. This framework represents a step toward autonomous, scalable, and industry-ready generative design solutions.

## Limitations

While the proposed AI-driven generative design framework demonstrates significant potential in producing lightweight and manufacturable mechanical structures, several limitations warrant consideration. First, the current implementation is confined to two-dimensional topology optimization scenarios, which restricts its applicability to real-world 3D mechanical components. Extending the methodology to fully three-dimensional domains will require increased computational resources and more complex reinforcement learning architectures. Second, the model assumes idealized material properties and boundary conditions that may not account for real-world variability in manufacturing or operating environments. Third, although the Signed Distance Field (SDF) filtering improves manufacturability, it does not yet fully integrate the constraints of specific additive manufacturing (AM) processes such as minimum feature resolution, support removal, or thermal deformation. Additionally, the reliance on the Topology Optimization Dataset (ToD) limits the diversity of load cases and structural configurations, potentially affecting model generalizability. Furthermore, the current reward function focuses mainly on structural compliance and material usage, with limited consideration for broader sustainability goals like recyclability or embodied carbon footprint. Finally, while PPO-based learning offers stability, it may suffer from slow convergence or suboptimal exploration in highly complex design spaces. Future enhancements will address these limitations to improve scalability, realism, and sustainability in intelligent structural design systems.

## Ethics statements

None.

## Related research article

None.

## CRediT authorship contribution statement

**Keerthi Kumar N:** Conceptualization, Methodology, Formal analysis, Writing – review & editing. **Manasa C M:** Supervision, Project administration. **Pavan Kumar B K:** Writing – original draft, Software. **Manish Bali:** Writing – review & editing.

## Declaration of competing interest

The authors declare that they have no known competing financial interests or personal relationships that could have appeared to influence the work reported in this paper.

## Data Availability

Data will be made available on request.
